# Bi-Lipschitz embeddings of the space of unordered $m$-tuples with a partial transportation metric

**DOI:** 10.1007/s00208-024-02831-x

**Published:** 2024-03-19

**Authors:** David Bate, Ana Lucía Garcia Pulido

**Affiliations:** 1 Mathematics Institute, University of Warwick, Coventry, CV4 7AL UK; 2 Division of Computing Science and Mathematics, University of Stirling, Stirling, FK9 4LA UK

## Abstract

Let $\Omega \subset {\mathbb {R}}^n$ be non-empty, open and proper. This paper is concerned with $Wb_p(\Omega )$, the space of *p*-integrable Borel measures on $\Omega$ equipped with the *partial* transportation metric introduced by Figalli and Gigli that allows the creation and destruction of mass on $\partial \Omega$. Alternatively, we show that $Wb_p(\Omega )$ is isometric to a subset of Borel measures with the ordinary Wasserstein distance, on the one point completion of $\Omega$ equipped with the shortcut metric

$$\begin{aligned} \delta (x,y)= \min \{\Vert x-y\Vert , {\text {dist}}(x,\partial \Omega )+{\text {dist}}(y,\partial \Omega )\}. \end{aligned}$$

In this article we construct bi-Lipschitz embeddings of the set of unordered *m*-tuples in $Wb_p(\Omega )$ into Hilbert space. This generalises Almgren’s bi-Lipschitz embedding theorem to the setting of optimal partial transport.

## Introduction

A striking variety of problems in geometry, analysis, combinatorics and a vast number of applications can be neatly formulated in terms of measures and their comparison using transportation metrics. The prototypical transportation metric is the *p-Wasserstein distance* [2]. This is defined between two Borel measures of the same total mass on a metric space (*X*, *d*) by

1 $$\begin{aligned} W_p(\mu ,\nu ) = \inf _{\gamma } \left( \int _{X\times X} d(x,y)^p \, \textrm{d}\gamma (x,y)\right) ^{\frac{1}{p}}, \end{aligned}$$

where $p\ge 1$ and the infimum is taken over all measures $\gamma$ on $X\times X$ with coordinate projections $\pi _1\gamma =\mu$ and $\pi _2\gamma =\nu$. The resulting metric space of *p*-integrable probability measures equipped with $W_p$ is denoted by $W_p(X)$ (see Definition 2.1).

A drawback of the Wasserstein distance is the requirement that the compared measures must have the same total mass. Recently emerging theories of *optimal partial transport* pertain to the transportation of measures without a mass constraint [8, 16, 20]. This article concerns the following formulation due to Figalli and Gigli [11].

Let $\Omega$ be an open non-empty proper subset of *X*. For *measures* $\mu$ and $\nu$ on $\Omega$, one defines $Wb_p(\mu ,\nu )$ as in (1), but the infimum is taken over measures $\gamma$ on ${\overline{\Omega }} \times {\overline{\Omega }}$ with

$$\begin{aligned} \pi _1 \gamma |_{\Omega }=\mu \quad \text {and} \quad \pi _2 \gamma |_{\Omega }=\nu . \end{aligned}$$

The resulting metric space of *p*-integrable measures, equipped with $Wb_p$, will be denoted by $Wb_p(X)$ (see Definition 3.1).

The key property of $Wb_p$ is that $\partial \Omega$ can be used to destroy or create mass, at a cost of transporting it to or from $\partial \Omega$. This allows measures of different total masses to be compared and hence one can construct a metric space consisting of *all* measures, instead of restricting to probability measures. Understanding the interplay between transportation metrics and $\partial \Omega$ is motivated by solving evolution equations with Dirichlet boundary conditions from gradient flows [11, 21]. The metric $Wb_p$ has found further applications such as obtaining new comparison principles for viscosity solutions [13].

A natural approach to study a metric space is to embed it into a well known space, such as a Euclidean or Banach space, as this allows the metric space to inherit geometric properties of the ambient space. Recall that the *distortion* of an injective map *f* between two metric spaces is ${\text {Lip}}(f)\cdot {\text {Lip}}(f^{-1})$, where ${\text {Lip}}(f)$ is the Lipschitz constant of *f*; *f* is *bi-Lipschitz* if it has finite distortion. Since bi-Lipschitz embeddings preserve relative distances, they are central to analysis and metric geometry [18] and have applications to algorithm design [14].

Due to the prominence of the Wasserstein spaces in various areas of mathematics, their embeddability has attracted much attention. The non-embeddability (into $L^1$) of $W_1$ over various discrete metric spaces [5] such as the planar grid [19] and Hamming cube [15] is known, as is the non-embeddability of $W_p({\mathbb {R}}^3)$ for $p\ge 1$ [4]. The interest in bi-Lipschitz embeddings of the Wasserstein spaces dates back to the work of Almgren [1, 9], forming the foundations of his celebrated partial regularity theorem for area minimising currents. Almgren proved that, for any $m\in {\mathbb {N}}$, the set of unordered $m$-tuples of points in ${\mathbb {R}}^n$,

$$\begin{aligned} \mathcal {A}_m({\mathbb {R}}^n) = \left\{ \sum _{i=1}^m[\![x_i]\!]: x_i\in {\mathbb {R}}^n\ \forall 1\le i \le m\right\} \end{aligned}$$

equipped with $W_2$, bi-Lipschitz embeds into some Euclidean space (see Theorem 2.3). Here and throughout, $[\![x ]\!]$ will denote the Dirac mass at *x*.

In this article we generalise Almgren’s embedding to $Wb_2(\Omega )$.

### Theorem 1.1

For $n\in {\mathbb {N}}$, let $\Omega \subset {\mathbb {R}}^n$ be non-empty, open and proper. The space $(\mathcal {B}_m(\Omega ),Wb_2)$ of unordered tuples of at most $m$ points bi-Lipschitz embeds into Hilbert space. The distortion of our embedding is at most $cm^{n+5/2}$, for some constant $c\ge 1$.

In general, $Wb_p(\Omega )$ is not a doubling metric space and hence cannot be bi-Lipschitz embedded into any Euclidean space, see Lemma 3.8. Therefore Hilbert space^1^ becomes the natural target for an embedding. Note that, since we are not constrained to comparing measures of the same total mass, in Theorem 1.1 we consider unordered tuples of *at most* $m$-points.

To prove Theorem 1.1, we first show, for $\Omega \subset X$, that $Wb_p(\Omega )$ isometrically embeds into the ordinary *p*-Wasserstein space of measures on $(\Omega ^*,\delta )$, where $\Omega ^*$ is the one point completion of $\Omega$ equipped with the shortcut metric

$$\begin{aligned} \delta (x,y)= \min \{\Vert x-y\Vert , {\text {dist}}(x,\partial \Omega )+{\text {dist}}(y,\partial \Omega )\} \end{aligned}$$

for every $x,y\in \Omega$ (see Lemma 3.3). This embedding maps $\mathcal {B}_m(\Omega )$ to $\mathcal {A}_m(\Omega ^*)$ and so, in order to prove Theorem 1.1, it remains to construct a bi-Lipschitz embedding of $\mathcal {A}_m(\Omega ^*)$ into Hilbert space.

We do this, for $\Omega \subset {\mathbb {R}}^n$, by considering a Whitney decomposition ${\mathcal {C}}$ of $\Omega$ into cubes. This decomposition is chosen such that, inside any cube $Q\in {\mathcal {C}}$, the shortcut metric equals the Euclidean metric and consequently

2 $$\begin{aligned} \mathcal {A}_m(Q,\delta ) = \mathcal {A}_m(Q,\Vert \ \Vert ). \end{aligned}$$

In particular, Almgren’s theorem gives an embedding of each $\mathcal {A}_m(Q)$ into some Euclidean space. Despite the fact that any measure can be written as a sum of measures supported on cubes in ${\mathcal {C}}$, the construction of the required bi-Lipschitz embedding of $\mathcal {A}_m(\Omega ^*)$ cannot be obtained simply by restricting to cubes. Indeed, $W_p$ may not even be defined between the restriction of two measures to a cube; even when it is, simple examples show that the optimal transport of the restricted measures may be incomparable to the optimal transport of the original measures.

Our approach uses (2) as the starting point to determine the optimal transport of measures between different cubes, see Sect. 4. From this analysis we construct a bi-Lipschitz embedding of $\mathcal {A}_m(\Omega ^*)$ into the $\ell _2$-sum of infinitely many copies of $\mathcal {A}_m({\mathbb {R}}^{n+1})$, see Theorem 4.12. The proof of Theorem 1.1 is concluded in Sect. 5 by applying Almgren’s embedding to each term of the $\ell _2$-sum.

We mention an application of Theorem 1.1 to persistence homology. The space of *persistence barcodes* can be viewed as $\cup _m\mathcal {B}_m(U)$ for

$$\begin{aligned} U = \{ (x,y)\in {\mathbb {R}}^2: y> x\}, \end{aligned}$$

see [10]. Theorem 1.1 shows that the space of persistence barcodes with at most $m$-points can be bi-Lipschitz embedded into Hilbert space. This answers questions raised by Carrière and Bauer [6]. Prior to our results, it was known that $\mathcal {B}_m(U)$ coarsely embeds into Hilbert space [17]. In fact, Theorem 1.1 applies to the generalised persistence barcodes introduced in [7] whenever the ambient space is Euclidean. Our theorem also holds when $\mathcal {B}_m(\Omega )$ is equipped with any $W_p$ for $p\ge 1$; due to the equivalence of norms on ${\mathbb {R}}^m$, these metrics are all bi-Lipschitz equivalent.

Finally, we mention that the distortion of any embedding of $\mathcal {B}_m(\Omega )$ into Hilbert space, for $\Omega \subset {\mathbb {R}}^n$, must necessarily converge to $\infty$ as $m$ does, see Remark 5.3.

## Wasserstein distance and Almgren’s embedding

Let (*X*, *d*) be a complete and separable metric space. We write ${\mathcal {M}}(X)$ for the set of Borel measures on *X* and ${\mathcal {P}}(X)$ for the set of Borel probability measures on *X*. The Wasserstein space is defined as follows [2, 3].

### Definition 2.1

For $\mu ,\nu \in {\mathcal {M}}(X)$ and $p \in [1,\infty )$ define

$$\begin{aligned} W_p(\mu ,\nu ) = \inf _{\gamma } \left( \int _{X\times X} d(x,y)^p \, \textrm{d}\gamma (x,y)\right) ^{\frac{1}{p}}, \end{aligned}$$

where the infimum is taken over all *couplings* $\gamma \in {\mathcal {M}}(X\times X)$ with coordinate projections $\pi _1\gamma =\mu$ and $\pi _2\gamma =\nu$. Note that $W_p(\mu ,\nu )<\infty$ only if $\mu (X)=\nu (X)$ as otherwise there does not exist a $\gamma$ as in Definition 2.1.

Let ${\mathcal {P}}_p(X)$ be those $\mu \in {\mathcal {P}}(X)$ with

$$\begin{aligned} \int _{X} d(x,x_0)^p \, \textrm{d}\mu (x) <\infty \end{aligned}$$

for some (equivalently all) $x_0\in X$. Then $W_p$ defines a metric on ${\mathcal {P}}_p(X)$. Analogous statements hold for the case $p=\infty$, where the $L^p$ integral is replaced by an essential supremum. We write $W_p(X)$ for the set ${\mathcal {P}}_p(X)$ equipped with $W_p$.

### Definition 2.2

For $m\in {\mathbb {N}}$, define the space of *unordered m-tuples*

$$\begin{aligned} \mathcal {A}_m(X) = \left\{ \sum _{i=1}^m[\![x_i]\!]: x_i\in X\ \forall 1\le i \le m\right\} , \end{aligned}$$

equipped with $W_2$. Note that, on $\mathcal {A}_m(X)$, $W_2$ equals

$$\begin{aligned} W_2(p,q) = \min _{\sigma \in \Sigma _m} \sqrt{\sum _{i=1}^md(p_i,q_{\sigma (i)})^2}, \end{aligned}$$

where $$p=\sum _{i=1}^m[\![p_i]\!]$$ and $$q=\sum _{i=1}^m[\![q_i]\!]$$.

A fundamental step in Almgren’s study of area minimising currents was the following bi-Lipschitz embedding.

### Theorem 2.3

(Almgren, Theorem 2.1 [9]) For every $m\in {\mathbb {N}}$ there exists an $N\in {\mathbb {N}}$ and a bi-Lipschitz embedding $\xi :\mathcal {A}_m({\mathbb {R}}^n) \rightarrow {\mathbb {R}}^N$. By inspecting the proof one sees that $\xi (0)=0$ and, for all $p,q \in \mathcal {A}_m({\mathbb {R}}^n)$,

$$\begin{aligned} \frac{W_2(p,q)}{cm^{n+1}} \le \Vert \xi (p)-\xi (q)\Vert \le W_2(p,q) \end{aligned}$$

for a constant $$c\ge 1$$.

## Optimal partial transport and the shortcut metric

The transportation metric $Wb$ introduced by Figalli and Gigli [11] is defined between two Borel measures. Originally defined for open and bounded $\Omega \subset {\mathbb {R}}^n$, we state the natural generalisation of $Wb$ to complete and separable metric spaces (*X*, *d*) (the proof of the triangle inequality is identical).

### Definition 3.1

Let $\Omega \subset X$ be proper and non-empty. For $\mu ,\nu \in {\mathcal {M}}(\Omega )$ and $p \in [1,\infty )$ define

$$\begin{aligned} Wb_p(\mu ,\nu ) = \inf _{\gamma } \left( \int _{X\times X} d(x,y)^p \, \textrm{d}\gamma (x,y)\right) ^{\frac{1}{p}}, \end{aligned}$$

where the infimum is taken over all *couplings* $\gamma \in {\mathcal {M}}(X\times X)$ with $\pi _1\gamma |_\Omega =\mu$ and $\pi _2\gamma |_\Omega =\nu$. Then $Wb_p$ defines a metric on

$$\begin{aligned} {\mathcal {M}}b_p(\Omega ):=\{\mu \in {\mathcal {M}}(\Omega ):Wb_p(\mu ,0)<\infty \}. \end{aligned}$$

Analogous statements hold for the case $p=\infty$, where the $L^p$ integral is replaced by an essential supremum.

We write $Wb_p(\Omega )$ for the set ${\mathcal {M}}b_p(\Omega )$ equipped with $Wb_p$. We also write $Wb^1_p(\Omega )$ for the set of $\mu \in {\mathcal {M}}b_p(\Omega )$ with $\mu (\Omega )\le 1$, equipped with $Wb_p$.

The first step in our proof of Theorem 1.1 is to show an equivalence between $Wb^1_p(\Omega )$ and $W_p(\Omega ^*)$, for $\Omega ^*$ the *shortcut* metric space, defined as the one point completion of $\Omega$ via its complement.

### Definition 3.2

For $\Omega \subset X$ non-empty and proper, let $\Omega ^*= \Omega \cup \{\partial \}$. For $x,y\in \Omega$ define

$$\begin{aligned} \delta (x,y)= \min \{\Vert x-y\Vert , {\text {dist}}(x,X{\setminus } \Omega )+{\text {dist}}(y,X{\setminus } \Omega )\} \end{aligned}$$

and $\delta (x,\partial ) = {\text {dist}}(x,X{\setminus } \Omega )$. Then $\delta$ defines a metric on $\Omega ^*$.

Profeta and Sturm [21, Remark 1.9] mention that $Wb_1^1(\Omega )$ isometrically embeds into $W_1(\Omega ^*)$, and give an example showing that their embedding is not an isometry for $p>1$. We show that there exists an isometric embedding of $Wb_p^1(\Omega )$ into $2W_p(\Omega ^*)$ for any $p\ge 1$. Here we write $2W_p(\Omega ^*)$ for the space of measures with total mass equal to 2.

### Lemma 3.3

Let *X* be a separable metric space and $\Omega \subset X$ be non-empty and proper. For any $p\ge 1$, the map

$$\begin{aligned} Wb_p^1(\Omega )&\rightarrow 2W_p(\Omega ^*)\\ \iota (\mu )&= \mu + (2-\mu (\Omega ))[\![\partial ]\!], \end{aligned}$$

is an isometric embedding.

### Proof

Given a coupling for $\mu ,\nu$ we use it to construct a coupling for $\iota (\mu ),\iota (\nu )$ and vice versa.

First let $\mu ,\nu \in Wb_p^1(\Omega )$ and suppose that $\gamma \in {\mathcal {M}}(X\times X)$ is a coupling for $\mu$ and $\nu$ in $Wb_p(\Omega )$. Let $\pi _\partial (x)=\partial$ for all $x\in X$ and define $\gamma '\in {\mathcal {M}}(\Omega ^*\times \Omega ^*)$ as

$$\begin{aligned} \gamma '&= \gamma |_{\Omega \times \Omega } + (\pi _\partial \times {\text {id}})_{\#} \gamma |_{X{\setminus } \Omega \times \Omega } + ({\text {id}} \times \pi _\partial )_{\#} \gamma |_{ \Omega \times X{\setminus } \Omega }\\&\qquad +(2-[\gamma (\Omega \times \Omega ) +\gamma (X{\setminus } \Omega \times \Omega ) + \gamma (\Omega \times X{\setminus } \Omega )])[\![(\partial ,\partial ) ]\!]. \end{aligned}$$

For notational convenience, we let $\kappa$ denote the coefficient of $[\![(\partial ,\partial ) ]\!]$ in this expression. Then

$$\begin{aligned} \pi _1 \gamma '&= \pi _1 (\gamma |_{\Omega \times \Omega }) + \gamma (X{\setminus } \Omega \times \Omega )[\![\partial ]\!]+ \pi _1(\gamma |_{\Omega \times X{\setminus } \Omega }) + \kappa [\![\partial ]\!]\\&= \pi _1 (\gamma |_{\Omega \times \Omega }) + \pi _1(\gamma |_{\Omega \times X{\setminus } \Omega }) + (2-[\gamma (\Omega \times \Omega )+\gamma (\Omega \times X{\setminus } \Omega )])[\![\partial ]\!]\\&= \pi _1(\gamma |_{\Omega \times X}) + (2-\gamma (\Omega \times X))[\![\partial ]\!]\\&= \mu + (2-\mu (X))[\![\partial ]\!]= \iota (\mu ). \end{aligned}$$

Similarly, by symmetry, $\pi _2\gamma ' = \iota (\nu )$. Thus $\gamma '$ is a coupling of $\iota (\mu )$ and $\iota (\nu )$ in $W_p(\Omega ^*)$. Moreover,

3 $$\begin{aligned} \int \delta (x,y)^p\, \textrm{d}\gamma '(x,y)&= \int _{\Omega \times \Omega } \delta (x,y)^p \, \textrm{d}\gamma (x,y)+ \int _{X{\setminus } \Omega \times \Omega } \delta (\partial ,y)^p\, \textrm{d}\gamma (x,y)\nonumber \\&\quad + \int _{\Omega \times X{\setminus } \Omega } \delta (x,\partial )^p d\gamma (x,y) +\kappa \delta (\partial ,\partial )^p\nonumber \\&\le \int _{\Omega \times \Omega } d(x,y)^p \, \textrm{d}\gamma (x,y) + \int _{X{\setminus } \Omega \times \Omega } d(x,y)^p \, \textrm{d}\gamma (x,y)\nonumber \\&\quad + \int _{\Omega \times X{\setminus } \Omega } d(x,y)^p\, \textrm{d}\gamma (x,y)\nonumber \\&=\int d(x,y)^p\, \textrm{d}\gamma (x,y). \end{aligned}$$

Therefore,

$$\begin{aligned} W_p(\iota (\mu ),\iota (\nu )) \le Wb_p(\mu ,\nu ). \end{aligned}$$

Conversely, let $\gamma$ be a coupling for $\iota (\mu )$ and $\iota (\nu )$ in $W_p(\Omega ^*)$. Define the closed set

$$\begin{aligned}E=\{(x,y)\in \Omega \times \Omega : \delta (x,y) = d(x,y)\}.\end{aligned}$$

Fix $\epsilon >0$ and for each $x\in \Omega$, let $c(x)\in X{\setminus } \Omega$ with

$$\begin{aligned} d(x,c(x)) \le (1+\epsilon ){\text {dist}}(x,X{\setminus } \Omega ). \end{aligned}$$

Since *X* is separable, *c* may be chosen to be a Borel function with countable image. Let $c_1=({\text {id}}\times c)\circ \pi _1$ and $c_2=(c\times {\text {id}})\circ \pi _2$ and define

4 $$\begin{aligned} \gamma ' = \gamma |_E + (c_1)_{\#} \gamma |_{(\Omega \times \Omega ^*){\setminus } E} + (c_2)_{\#} \gamma |_{(\Omega ^*\times \Omega ){\setminus } E} \in {\mathcal {M}}(X\times X). \end{aligned}$$

Note that, since $\pi _1((c_1)_{\#}\gamma )$ is supported on $X{\setminus } \Omega$, its restriction to $\Omega$ equals 0. Therefore,

$$\begin{aligned} (\pi _1\gamma ')|_\Omega = (\pi _1\gamma |_E)|_\Omega + (\pi _1\gamma |_{(\Omega \times \Omega ^*){\setminus } E})|_\Omega + 0 = (\pi _1 \gamma )|_\Omega = \mu . \end{aligned}$$

Similarly, by symmetry, $(\pi _2\gamma ')|_\Omega = \nu$. Hence $\gamma$ is a coupling for $\mu$ and $\nu$ in $Wb_p(\Omega )$.

Now, for any $(x,y)\in (\Omega \times \Omega ){\setminus } E$,

$$\begin{aligned} d(x,c(x))^p + d(c(y),y)^p \le (1+\epsilon )^p(\delta (x,\partial )^p + \delta (\partial ,y)^p) \le (1+\epsilon )^p\delta (x,y)^p. \end{aligned}$$

Therefore,

5 $$\begin{aligned} \int _{X\times X} d(x,y)^p\, \textrm{d}\gamma '(x,y)&= \int _E d(x,y)^p\, \textrm{d}\gamma (x,y) + \int _{(\Omega \times \Omega ^*){\setminus } E} d(x,c(x))^p\, \textrm{d}\gamma (x,y)\nonumber \\&\quad + \int _{(\Omega ^*\times \Omega ){\setminus } E} d(c(y),y)^p\, \textrm{d}\gamma (x,y)\nonumber \\&\le \int _E d(x,y)^p\, \textrm{d}\gamma (x,y) \nonumber \\&\quad + \int _{(\Omega ^*\times \Omega ^*){\setminus } E} (1+\epsilon )^p\delta (x,y)^p\, \textrm{d}\gamma (x,y)\nonumber \\&\le (1+\epsilon )^p\int _{\Omega ^*\times \Omega ^*} \delta (x,y)^p\, \textrm{d}\gamma (x,y). \end{aligned}$$

Since $\epsilon >0$ is arbitrary, this shows that

$$\begin{aligned}W_p(\iota (\mu ),\iota (\nu )) \ge Wb_p(\mu ,\nu ).\end{aligned}$$

$$\square $$

### Remark 3.4

After the first version of this article appeared, we were made aware that the statement of Lemma 3.3, for the case $\Omega =U$ as defined in our introduction, appears in the work of Divol and Lacombe [10, Proposition 3.15]. Note that our proof does not rely on the existence of unique closest points in $\partial \Omega$, whilst the one in [10] does. However, a flaw in their argument makes the proof incorrect even for the case of $\Omega =U$.

Central to their proof is the definition of a measure ${\tilde{\pi }}'$ and the claim that it is a coupling of ${\tilde{\mu }}$ and ${\tilde{\nu }}$ in $W_p(\Omega ^*)$ (using the variables of [10, Lemma 3.17]). Using this they derive [10, Equation (3.8)] from which the proof is concluded. However, examples such as [21, Remark 1.9] show this equation to be false. Moreover, this equation would imply that $\delta =d$ in $\Omega$. These contradictions originate in the fact that ${\tilde{\pi }}'$ is not a coupling of ${\tilde{\mu }}$ and ${\tilde{\nu }}$, which can be verified by comparing the total measure of ${\tilde{\pi }}'$ to that of ${\tilde{\mu }},{\tilde{\nu }}$ or ${\tilde{\pi }}$.

Since the map

$$\begin{aligned} 2W_p(\Omega ^*)&\rightarrow W_p(\Omega ^*)\\ \mu&\mapsto \mu /2 \end{aligned}$$

has distortion $2^{1/p}$, we obtain the following corollary.

### Corollary 3.5

Let *X* be a separable metric space and $\Omega \subset X$ be non-empty and proper. Then $Wb_p^1(\Omega )$ bi-Lipschitz embeds into $W_p(\Omega ^*)$ with distortion 2.

The same proof as the one for Lemma 3.3 shows that the full space $Wb_p(\Omega )$ isometrically embeds into ${\mathcal {M}}(\Omega ^*)$.

### Lemma 3.6

Let *X* be a separable metric space and $\Omega \subset X$ non-empty and proper. For any $p\ge 1$,

$$\begin{aligned} Wb_p(\Omega )&\rightarrow ({\mathcal {M}}(\Omega ^*),W_p)\\ \iota '(\mu )&= \mu + \infty \cdot [\![\partial ]\!]\end{aligned}$$

is an isometric embedding.

### Remark 3.7

For any $$\mu \in {\mathcal {M}}b_p(\Omega )$$,

$$\begin{aligned} W_p(\iota '(\mu ),\infty \cdot [\![\partial ]\!])=Wb_p(\mu ,0)<\infty . \end{aligned}$$

Therefore, the triangle inequality for $W_p$ implies that $W_p$ is indeed a metric on the image of $\iota '$.

### Proof

(Proof of Lemma 3.6) If $\mu ,\nu \in {\mathcal {M}}b_p(\Omega )$ then

$$\begin{aligned} \gamma ' = \gamma |_{\Omega \times \Omega } + (\pi _\partial \times {\text {id}})_{\#} \gamma |_{X{\setminus } \Omega \times \Omega } + ({\text {id}} \times \pi _\partial )_{\#} \gamma |_{ \Omega \times X{\setminus } \Omega } +\infty \cdot [\![(\partial ,\partial )]\!]\end{aligned}$$

defines a coupling of $\iota '(\mu )$ and $\iota '(\nu )$. The calculation in (3) shows that

$$\begin{aligned}W_p(\iota '(\mu ),\iota '(\nu )) \le Wb_p(\mu ,\nu ).\end{aligned}$$

Conversely, if $\mu ,\nu \in {\mathcal {M}}b_p(\Omega )$, then $\gamma '$ as defined in (4) is a coupling for $\mu ,\nu$ and (5) shows that

$$\begin{aligned} W_p(\iota '(\mu ),\iota '(\nu )) \ge Wb_p(\mu ,\nu ).\end{aligned}$$

$$\square $$

### The shortcut metric space is not doubling

A metric space *X* is *doubling* if there exists $N\in {\mathbb {N}}$ such that each ball $B\subset X$ is covered by *N* balls of half the radius of *B*.

#### Lemma 3.8

For $n\ge 2$, let $\Omega \subset {\mathbb {R}}^n$ be non-empty and open such that ${\overline{\Omega }}$ is a proper subset of ${\mathbb {R}}^n$. Then for any $N\in {\mathbb {N}}$ and any sufficiently small $\epsilon >0$, there exist $y_1,\ldots ,y_N\in \Omega$ with $\delta (y_i,y_j)=\epsilon$ for each $i\ne j$. In particular, $\Omega ^*$ is not doubling.

#### Proof

Let $x\not \in {\overline{\Omega }}$ and $y\in \Omega$. For $N\in {\mathbb {N}}$, let $y_1,\ldots ,y_N\in \Omega$ lie on the circle centred on *x* of radius $\Vert x-y\Vert$ (such points exist since $\Omega$ is open). For each $1\le i\le N$, let $l'_i$ be the line segment connecting $y_i$ to *x* and let $l_i$ be the connected component of $l'_i\cap \Omega$ containing $y_i$. Since $x\not \in {\overline{\Omega }}$, there exists $\eta >0$ such that

$$\begin{aligned} \inf \{ \Vert z-z'\Vert : z\in l_i,\ z'\in l_j,\ i\ne j\} >\eta . \end{aligned}$$

Now, ${\text {dist}}(\cdot ,\partial \Omega )$ is continuous on each $l_i$ and converges to 0 as one travels along $l_i$ towards $\partial \Omega$. Therefore, for each sufficiently small $\epsilon >0$ and each $1\le i \le N$, there exists $z_i\in l_i$ with ${\text {dist}}(z_i,\partial \Omega )=\epsilon /2$. In particular, if $\epsilon <\eta$, then $\delta (z_i,z_j)=\epsilon$ for each $1\le i\ne j\le N$.

Finally, we see that $y_i\in B(y_1,\epsilon )$ for each $1\le j\le N$, but we require at least *N* balls of radius $\epsilon /4$ to cover $B(y_1,\epsilon )$. Since $N\in {\mathbb {N}}$ is arbitrary, $\Omega ^*$ cannot be doubling. $\square$

#### Remark 3.9

Lemma 3.8 is sharp in the following sense. If $\Omega =(-1,1)\subset {\mathbb {R}}$, then $\Omega ^*$ is bi-Lipschitz equivalent to a Euclidean circle. For any $n\in {\mathbb {N}}$, if $\Omega = {\mathbb {R}}^n{\setminus } \{0\}$, then $\Omega ^*$ is isometric to ${\mathbb {R}}^n$. In both of these cases, the conclusion of Lemma 3.8 fails.

Note that each Euclidean space is doubling and that the doubling property is preserved under taking subsets and bi-Lipschitz images. Therefore, if a metric space is bi-Lipschitz embeddable into some Euclidean space, it must necessarily be doubling.

#### Corollary 3.10

For $n\ge 2$ let $\Omega \subset {\mathbb {R}}^n$ be non-empty and open such that ${\overline{\Omega }}$ is a proper subset of ${\mathbb {R}}^n$. Then $\Omega ^*$ is not bi-Lipschitz embeddable into any Euclidean space.

### The space of unordered tuples of at most $m$ points

#### Definition 3.11

Let *X* be a metric space, $\Omega \subset X$ non-empty and proper and $m\in {\mathbb {N}}$. Define the *space of unordered tuples of at most m points* as

$$\begin{aligned} \mathcal {B}_m(\Omega ) = \bigcup _{k=1}^m\mathcal {A}_k(\Omega ), \end{aligned}$$

with the metric inherited from $Wb_2(\Omega )$.

This space is naturally identified with a subset of $\mathcal {A}_m(\Omega ^*)$.

#### Corollary 3.12

Let $m\in {\mathbb {N}}$. For any separable metric space *X* and non-empty and proper $\Omega \subset X$, $\mathcal {B}_m(\Omega )$ isometrically embeds into $\mathcal {A}_m(\Omega ^*)$ via the map

$$\begin{aligned} \sum _{i=1}^k [\![x_i]\!]\mapsto \sum _{i=1}^k [\![x_i]\!]+ (2m-k)[\![\partial ]\!]. \end{aligned}$$

#### Proof

Embed $\mathcal {B}_m(X)$ into $Wb^1_p(X)$ by $\mu \mapsto \mu /m$, apply Lemma 3.3, and then embed into $\mathcal {A}_m(\Omega ^*)$ by $\mu \mapsto m\mu$. $\square$

## A bi-Lipschitz description of $\mathcal {A}_m(\Omega ^*)$ in terms of $\mathcal {A}_m({\mathbb {R}}^{n+1})$

To construct the bi-Lipschitz embedding from Theorem 1.1, it would be natural to adapt the techniques from the proof of Theorem 2.3 to our setting. However, the proof of Theorem 2.3 strictly depends on both, the linear structure of ${\mathbb {R}}^n$ (in particular the existence of projections), and the compactness of the unit ball. Although $\Omega \subset {\mathbb {R}}^n$ as a set, $\delta$ bears no relationship to the linear structure of ${\mathbb {R}}^n$ and this fact prohibits the direct use of Almgren’s techniques. On the other hand, whilst it is possible to find a bi-Lipschitz embedding of $\Omega ^*$ into $\ell _2$ to gain a linear structure, this comes at the expense of compactness of the unit ball. Thus it is not possible to modify Almgren’s proof to our setting.

In order to prove Theorem 1.1 we will use a Whitney decomposition ${\mathcal {C}}$ of $\Omega$ into cubes

$$\begin{aligned} \Omega = \bigcup _{Q\in {\mathcal {C}}} Q\end{aligned}$$

(see Proposition 4.2) such that, within each $Q$, $\delta$ is given by $\Vert \cdot \Vert$. Consequently, $\mathcal {A}_m(Q,\delta )=\mathcal {A}_m(Q,\Vert \cdot \Vert )$. Theorem 2.3 then gives a bi-Lipschitz embedding of each $\mathcal {A}_m(Q,\delta )$ into ${\mathbb {R}}^N$ and it would be favourable to use these embeddings as “coordinate projections" to construct a global embedding into Hilbert space. Of course, the union of the $\mathcal {A}_m(Q)$ does not cover $\mathcal {A}_m(\Omega ^*)$ and therefore we cannot simply define coordinate projections by taking restrictions to each $Q$. Nevertheless, the fact that $\mathcal {A}_m(Q,\delta )=\mathcal {A}_m(Q,\Vert \cdot \Vert )$ enables us to construct a map $\phi ^*_Q:\mathcal {A}_m(\Omega ^*)\rightarrow \mathcal {A}_m({\mathbb {R}}^{n+1})$ which, roughly speaking, acts as a smooth projection to $\mathcal {A}_m(Q)$.

The main result of this section shows that the $\phi ^*_Q$ can be combined to define a bi-Lipschitz embedding of $\mathcal {A}_m(\Omega ^*)$ into the following metric space.

### Definition 4.1

Let ${\mathcal {C}}$ be a countable set and define

$$\begin{aligned} {\mathcal {T}}:=\sum _{Q\in {\mathcal {C}}} \mathcal {A}_m({\mathbb {R}}^{n+1}) \end{aligned}$$

to be the $\ell _2$-sum of copies of $\mathcal {A}_m({\mathbb {R}}^{n+1})$. That is, ${\mathcal {T}}$ consists of sequences

$$\begin{aligned} \sum _{Q\in {\mathcal {C}}} a_Q\end{aligned}$$

of elements of $\mathcal {A}_m({\mathbb {R}}^{n+1})$ for which

$$\begin{aligned} \sum _{Q\in {\mathcal {C}}}W_2^2(a_Q,0)<\infty , \end{aligned}$$

where $0=\sum _{i=1}^m[\![0]\!]$, equipped with the metric

$$\begin{aligned} \sqrt{\sum _{Q\in {\mathcal {C}}} W_2^2(a_Q,a'_Q)}. \end{aligned}$$

Once we have an embedding into ${\mathcal {T}}$, we will show that it is possible to find an embedding into $\ell _2$. Indeed, in Sect. 5, we apply Theorem 2.3 to each term in the definition of ${\mathcal {T}}$ to obtain a bi-Lipschitz embedding of ${\mathcal {T}}$ into $\ell _2$.

### A Whitney decomposition of $\Omega$

To construct the embedding into ${\mathcal {T}}$, we will use a Whitney decomposition of $\Omega$. For a cube $Q\subset {\mathbb {R}}^n$, let $l(Q)$ denote the side length of $Q$.

#### Proposition 4.2

(Appendix J [12]) Let $\Omega \subset {\mathbb {R}}^n$ be non-empty, open and proper. There exists a family of closed cubes ${\mathcal {C}}$ such that

1. $\cup {\mathcal {C}}= \Omega$ and the elements of ${\mathcal {C}}$ have disjoint interiors.
2. $$\sqrt{n} l(Q) \le {\text {dist}}(Q,\partial \Omega ) \le 4 \sqrt{n} l(Q)$$ for all $$Q\in {\mathcal {C}}$$.
3. If $Q,Q'\in {\mathcal {C}}$ and $Q\cap Q'\ne \emptyset$ then
  $$\begin{aligned} \frac{1}{4} \le \frac{l(Q)}{l(Q')} \le 4. \end{aligned}$$
  We say that $Q,Q'$ are *neighbours*.
4. Each $Q\in {\mathcal {C}}$ has at most $12^n$ neighbours.

A Whitney decomposition of $\Omega$ estimates which quantity attains the minimum in the definition of $\delta$.

#### Lemma 4.3

Let ${\mathcal {C}}$ be a Whitney decomposition of $\Omega \subset {\mathbb {R}}^n$, $Q,Q'\in {\mathcal {C}}$ and $x\in Q$ and $y\in Q'$. Then

6 $$\begin{aligned} \sqrt{n} l(Q) \le {\text {dist}}(x,\partial \Omega ) \le 5\sqrt{n} l(Q). \end{aligned}$$

If $Q,Q'$ are neighbours then

7 $$\begin{aligned} \delta (x,y) = \Vert x-y\Vert . \end{aligned}$$

If $Q,Q'$ are not neighbours then

8 $$\begin{aligned} \frac{l(Q)+l(Q')}{8} \le \delta (x,y) \le 5\sqrt{n}(l(Q)+l(Q')). \end{aligned}$$

#### Proof

The first inequality in (6) is implied by $\sqrt{n}l(Q)\le {\text {dist}}(Q,\partial \Omega )$. The second follows from the triangle inequality:

$$\begin{aligned} {\text {dist}}(x,\partial \Omega ) \le {\text {dist}}(Q,\partial \Omega ) + {\text {diam}}(Q) \le 4\sqrt{n}l(Q) + \sqrt{n}l(Q). \end{aligned}$$

Now suppose $Q,Q'$ are neighbours and let $z\in Q\cap Q'$. Then by (6),

$$\begin{aligned} {\text {dist}}(x,\partial \Omega ) + {\text {dist}}(y,\partial \Omega )&\ge \sqrt{n}(l(Q)+l(Q'))\\&\ge \Vert x-z\Vert + \Vert z-y\Vert \ge \Vert x-y\Vert , \end{aligned}$$

giving (7). On the other hand, suppose that $Q,Q'$ are not neighbours and $l(Q)\ge l(Q')$. Then $\Vert x-y\Vert \ge l(Q'')$ for $Q''$ a neighbour of $Q$. In particular

$$\begin{aligned} \Vert x-y\Vert \ge l(Q'')\ge \frac{l(Q)}{4} \ge \frac{l(Q)+l(Q')}{8}, \end{aligned}$$

giving the first inequality in (8). The second inequality follows from (6). $\square$

For the remainder of the paper we fix $m\in {\mathbb {N}}$, $\Omega \subset {\mathbb {R}}^n$ non-empty, open and proper and ${\mathcal {C}}$ a Whitney decomposition of $\Omega$ as in Proposition 4.2. We also fix ${\mathcal {T}}$ as in Definition 4.1.

### Constructing a coordinate system

To construct a bi-Lipschitz embedding of $\mathcal {A}_m(\Omega ^*)$ into ${\mathcal {T}}$, we define projections

$$\begin{aligned} \phi ^*_Q:\mathcal {A}_m(\Omega ^*)\rightarrow \mathcal {A}_m({\mathbb {R}}^{n+1}) \end{aligned}$$

that serve as a coordinate system for $\mathcal {A}_m(\Omega ^*)$. The embedding into ${\mathcal {T}}$ will then be defined as the $\ell _2$-sum of the $\phi ^*_Q$ (see Definition 4.6).

We begin with the construction of a function $\phi _Q$ that approximates the identity within a given $Q\in {\mathcal {C}}$, is supported on the neighbours of $Q$, and maintains bi-Lipschitz bounds with $\delta$. For $Q\in {\mathcal {C}}$ and $r>0$, we write $B(Q,r)$ for the *closed* *r*-neighbourhood of $Q$.

#### Lemma 4.4

For each $Q\in {\mathcal {C}}$ there exists a map

$$\begin{aligned}\phi _Q:\Omega \rightarrow {\mathbb {R}}^{n+1}\end{aligned}$$

such that

1. $$\phi _Q$$ is $$9\sqrt{n+1}$$-Lipschitz;
2. $\phi _Q(x)=0$ for all $x\not \in B(Q,l(Q)/4)$. In particular, $\phi _Q$ is supported on the neighbours of $Q$;
3. $$\Vert \phi _Q\Vert _\infty \le \sqrt{n+1}l(Q)$$;
4. For all $$x,y\in B(Q,l(Q)/8)$$,
  $$\begin{aligned}\Vert \phi _Q(x)-\phi _Q(y)\Vert =\Vert x-y\Vert ;\end{aligned}$$
5. The extension of $\phi _Q$ to $\Omega ^*$, defined by $\phi _Q(\partial )=0$, is $9\sqrt{n+1}$-Lipschitz with respect to $\delta$;
6. If $x\in B(Q,l(Q)/8)$ and $y\in \Omega ^*$, then
  $$\begin{aligned} \Vert \phi _Q(x)-\phi _Q(y)\Vert \ge \min \left\{ \frac{\Vert x-y\Vert }{2\sqrt{n}}, l(Q)\right\} . \end{aligned}$$

#### Proof

Fix $Q\in {\mathcal {C}}$ and let *c* be the centre of $Q$. For each $x\in \Omega$, let

$$\begin{aligned} \eta (x)= \max \left\{ 1-{\text {dist}}\left( x,B\left( Q, \frac{l(Q)}{8}\right) \right) \frac{8}{l(Q)},0\right\} . \end{aligned}$$

That is, $\eta$ is an $8/l(Q)$-Lipschitz function with $\Vert \eta \Vert _\infty =1$ that equals 1 on $B(Q,l(Q)/8)$ and 0 on $\Omega {\setminus } B(Q,l(Q)/4)$. We also set

$$\begin{aligned}\varphi (x)=(x-c,l(Q)) \in {\mathbb {R}}^{n+1},\end{aligned}$$

a 1-Lipschitz function satisfying $\Vert \varphi (x)\Vert \le \sqrt{n+1}l(Q)$ for all *x* in the support of $\eta$.

Define $\phi _Q= \eta \varphi$. Since $\phi _Q$ is a product of Lipschitz functions, the Lipschitz constant of $\phi _Q$ is bounded above by

$$\begin{aligned} {\text {Lip}}\varphi \Vert \eta \Vert _\infty + \sup \{\Vert \varphi (x)\Vert : x\in {\text {spt}} \eta \} {\text {Lip}}\eta \le 1 + \sqrt{n+1}l(Q) \frac{8}{l(Q)} \le 9\sqrt{n+1}. \end{aligned}$$

This demonstrates item 1. Items 2 to 4 are immediate.

To see item 5, first let $x\in \Omega ^*$ be such that $\phi _Q(x)\ne 0$. Then by item 2, $x\in Q'$ for $Q'$ a neighbour of $Q$, so that $l(Q')\ge l(Q)/4$. Therefore, by item 3,

$$\begin{aligned} \Vert \phi _{Q}(x)\Vert&\le \sqrt{n+1}\ l(Q)\\&\le 4\sqrt{n+1}\ l(Q')\\&\le 8{\text {dist}}(x,\partial \Omega ), \end{aligned}$$

using Eq. (6) for the final inequality. Thus

$$\begin{aligned} \Vert \phi _Q(x)\Vert \le 8{\text {dist}}(x,\partial \Omega ) \end{aligned}$$

holds for any $x\in \Omega ^*$ (including $x=\partial$). Therefore, by the triangle inequality, for any $x,y\in \Omega ^*$,

$$\begin{aligned} \Vert \phi _{Q}(x)-\phi _{Q}(y)\Vert \le 8\left( {\text {dist}}(x,\partial \Omega ) +{\text {dist}}(y,\partial \Omega )\right) . \end{aligned}$$

Combining this inequality with item 1 shows that $\phi _{Q}$ is $9\sqrt{n+1}$-Lipschitz with respect to $\delta$ on $\Omega ^*$.

Finally, to see item 6, first suppose that $y\not \in B(Q,l(Q)/4)$. Then by item 2,

$$\begin{aligned}\Vert \phi _{Q}(x)-\phi _{Q}(y)\Vert = \Vert \varphi (x)\Vert \ge l(Q),\end{aligned}$$

so that item 6 holds in this case.

In the case $y\in B(Q,l(Q)/4)$ we will show that

9 $$\begin{aligned} \Vert \phi _{Q}(x)-\phi _{Q}(y)\Vert \ge \frac{\Vert x-y\Vert }{2\sqrt{n}}, \end{aligned}$$

completing the proof of item 6. To this end, note that

$$\begin{aligned} \Vert y-c\Vert \le \sqrt{n}\frac{l(Q)}{2} + \frac{l(Q)}{4} \le \sqrt{n}l(Q). \end{aligned}$$

Therefore, by considering the first component of $\phi _Q$, we see that

$$\begin{aligned} \Vert \phi _Q(x)-\phi _Q(y)\Vert&\ge \Vert (x-c) -\eta (y)(y-c)\Vert \\&\ge \Vert x-y\Vert -(1-\eta (y))\Vert y-c\Vert \\&\ge \Vert x-y\Vert -\sqrt{n}(1-\eta (y))l(Q). \end{aligned}$$

Thus, if

10 $$\begin{aligned} \sqrt{n}(1-\eta (y))l(Q) \le \frac{\Vert x-y\Vert }{2}, \end{aligned}$$

then (9) holds. On the other hand, if (10) does not hold, then by considering the final component of $\phi$, we have

$$\begin{aligned} \Vert \phi _Q(x)-\phi _Q(y)\Vert \ge (1-\eta (y))l(Q) \ge \frac{\Vert x-y\Vert }{2\sqrt{n}}, \end{aligned}$$

giving (9). $$\square $$

The pushforwards under each $\phi _Q$ define our coordinate projections on $\mathcal {A}_m(\Omega )$.

#### Definition 4.5

For every $Q\in {\mathcal {C}}$, define $\phi ^*_Q$ to be the pushforward under $\phi _Q$. That is,

$$\begin{aligned} \phi ^*_Q:\mathcal {A}_m(\Omega ^*)&\rightarrow \mathcal {A}_m({\mathbb {R}}^{n+1})\\ \sum _{i=1}^m[\![p_i ]\!]&\mapsto \sum _{i=1}^m[\![\phi _Q(p_i) ]\!]. \end{aligned}$$

Recall the construction of ${\mathcal {T}}$ from Definition 4.1.

#### Definition 4.6

Define the embedding $\phi ^*$ by

$$\begin{aligned} \mathcal {A}_m(\Omega ^*)&\rightarrow {\mathcal {T}}\\ \phi ^*&= \sum _{Q\in {\mathcal {C}}} \phi ^*_Q\end{aligned}$$

This is well defined since each $\phi _Q$ is supported on the neighbours of $Q$, so that each $x\in \Omega$ is contained in the support of at most $12^n$ of the $\phi _Q$.

### $\phi ^*$ is bi-Lipschitz

In this section we show that $\phi ^*$ is a bi-Lipschitz embedding, beginning by showing that it is Lipschitz.

For $p\in (\Omega ^*)^m$ and $S\subset \Omega ^*$, let

$$\begin{aligned}p^{-1}(S)=\{1\le k\le m: p_k\in S\}.\end{aligned}$$

From now on we use the notation $\sigma q$ to denote the element of $({\mathbb {R}}^n)^m$ arising from the natural action of the symmetric group $\Sigma _m$ on $({\mathbb {R}}^n)^m$: $(\sigma q)_i = q_{\sigma (i)}$ for each $1\le i \le m$.

#### Lemma 4.7

For any $$p,q\in \mathcal {A}_m(\Omega ^*)$$,

$$\begin{aligned} \sum _{Q\in {\mathcal {C}}}W_2(\phi ^*_Q(p),\phi ^*_Q(q))^2 \le c_0 W_2^2(p,q), \end{aligned}$$

where $c_0\ge 1$ depends only upon *n*.

#### Proof

Fix $p,q\in (\Omega ^*)^m$ and let $Q\in {\mathcal {C}}$ and $\sigma \in \Sigma _m$. Set

$$\begin{aligned} J_{Q}^{\sigma }=p^{-1}(B(Q, l(Q)/4))\cup (\sigma q)^{-1}(B(Q, l(Q)/4)), \end{aligned}$$

so that, by Lemma 4.4 item 2,

$$\begin{aligned} \sum _{k=1}^m\Vert \phi _{Q}(p_k)-\phi _{Q}(q_{\sigma (k)})\Vert ^2 = \sum _{k\in J_Q^\sigma }\Vert \phi _{Q}(p_k)-\phi _{Q}(q_{\sigma (k)})\Vert ^2. \end{aligned}$$

Applying Lemma 4.4 item 5 gives

$$\begin{aligned} \sum _{k=1}^m\Vert \phi _{Q}(p_k)-\phi _{Q}(q_{\sigma (k)})\Vert ^2 \le 9^2(n+1) \sum _{k\in J_Q^\sigma } \delta (p_k,q_{\sigma (k)})^2. \end{aligned}$$

Therefore

$$\begin{aligned} \sum _{Q\in {\mathcal {C}}}\min _{\sigma \in \Sigma _m} \sum _{k=1}^m\Vert \phi _{Q}(p_k)-\phi _{Q}(q_{\sigma (k)})\Vert ^2 \le 9^2(n+1)\sum _{Q\in {\mathcal {C}}} \min _{\sigma \in \Sigma _m}\sum _{k\in J_Q^\sigma } \delta (p_k,q_{\sigma (k)})^2. \end{aligned}$$

Further,

$$\begin{aligned} \sum _{Q\in {\mathcal {C}}}\min _{\sigma \in \Sigma _m} \sum _{k\in J_Q^\sigma } \delta (p_k,q_{\sigma (k)})^2&\le \min _{\sigma \in \Sigma _m}\sum _{Q\in {\mathcal {C}}} \sum _{k\in J_Q^\sigma } \delta (p_k,q_{\sigma (k)})^2\\&\le \min _{\sigma \in \Sigma _m} 2\cdot 12^n \sum _{k=1}^m\delta (p_k,q_{\sigma (k)})^2, \end{aligned}$$

since $B(Q,l(Q)/4)$ is contained within the union of the neighbours of $Q$. The result follows for $c_0= 2\cdot 9^2 \cdot 12^n (n+1)$. $\square$

To prove the lower Lipschitz bound, we fix the following notation until the end of the section.

#### Notation 4.8

Fix $p,q\in (\Omega ^*)^m$ and, for every $Q\in {\mathcal {C}}$, let $\sigma _Q\in \Sigma _m$ be such that

11 $$\begin{aligned} \sum _{k=1}^m\Vert \phi _{Q}(p_k)-\phi _{Q}(q_{\sigma _Q(k)})\Vert ^2 = W_2(\phi ^*_Q(p),\phi ^*_Q(q))^2. \end{aligned}$$

Let $Q\in {\mathcal {C}}$. For integer $0\le r \le 2m$, the annuli

$$\begin{aligned} Q^r=B\left( Q,\frac{r+1}{3m} \frac{l(Q)}{8}\right) {\setminus } B\left( Q,\frac{r}{3m} \frac{l(Q)}{8}\right) \end{aligned}$$

are disjoint and so there exists $0\le r \le 2m$ such that

12 $$\begin{aligned} p^{-1}(Q^{r})\cup (\sigma _Qq)^{-1}(Q^{r})=\emptyset . \end{aligned}$$

Set

$$\begin{aligned} {\widehat{Q}}= B\left( Q,\frac{r}{3m} \frac{l(Q)}{8}\right) . \end{aligned}$$

Note that ${\widehat{Q}}$ is contained within the union of the neighbours of $Q$.

Let $c_1=(48\sqrt{n})^{-1}$ and define ${\mathcal {C}}'$ to be the set of $Q\in {\mathcal {C}}$ for which

13 $$\begin{aligned} W_2(\phi ^*_Q(p),\phi ^*_Q(q)) < c_1 \frac{l(Q)}{m}. \end{aligned}$$

Set

$$\begin{aligned} E = \bigcup _{Q\in {\mathcal {C}}'} {\widehat{Q}}. \end{aligned}$$

To obtain a lower bound of

14 $$\begin{aligned} \sum _{Q\in {\mathcal {C}}}W_2(\phi ^*_Q(p),\phi ^*_Q(q))^2 \end{aligned}$$

in terms of $W_2^2(p,q)$, we will construct a $\tau \in \Sigma _m$ for which $\sum _{i=1}^m\delta (p_i,q_{\tau (i)})^2$ is comparable to (14). A first attempt to do this may be, for each $Q\in {\mathcal {C}}$ and each $i\in p^{-1}(Q)$, to define $\tau (i)=\sigma _Q(i)$. Of course, a $\tau$ defined in this way need not be injective, for example if there exist $Q\ne Q'\in {\mathcal {C}}$ and $i\ne j$ such that $q_{\sigma _Q(i)} = q_{\sigma _{Q'}(j)}$. Nonetheless, we will show that it is possible to construct a permutation for the cubes in ${\mathcal {C}}'$. Indeed, we now show that conditions (12) and (13) ensure that, for each $Q\in {\mathcal {C}}'$, $p_i\in \widehat{Q}$ if and only if $q_{\sigma _Q(i)}\in {\widehat{Q}}$: (12) provides a moat surrounding ${\widehat{Q}}$ and (13) ensures that the distance between $p_i$ and $q_{\sigma _Q(i)}$ is less than the width of the moat.

#### Lemma 4.9

For any $$Q\in {\mathcal {C}}'$$,

15 $$\begin{aligned} p^{-1}({\widehat{Q}}) = (\sigma _Qq)^{-1}({\widehat{Q}}) \end{aligned}$$

and

16 $$\begin{aligned} \Vert p_k-q_{\sigma _Q(k)}\Vert =\Vert \phi _Q(p_k)-\phi _Q(q_{\sigma _Q(k)})\Vert \quad \forall k\in p^{-1}({\widehat{Q}}). \end{aligned}$$

Moreover, if $R\in {\mathcal {C}}'$ with $l(R)\le l(Q)$,

17 $$\begin{aligned} p^{-1}({\widehat{Q}} \cap {\widehat{R}})= (\sigma _R q)^{-1}({\widehat{Q}} \cap {\widehat{R}}) \end{aligned}$$

#### Proof

For any $k\in p^{-1}({\widehat{Q}})$, (13) and Lemma 4.4 item 6 imply

$$\begin{aligned} \min \left\{ \frac{\Vert p_k-q_{\sigma _Q(k)}\Vert }{2\sqrt{n}},l(Q)\right\} < c_1\frac{l(Q)}{m}. \end{aligned}$$

In particular,

18 $$\begin{aligned} \Vert p_k-q_{\sigma _Q(k)}\Vert < \frac{l(Q)}{24m}. \end{aligned}$$

Therefore (12) implies that $q_{\sigma _Q(k)}\in {\widehat{Q}}$. By symmetry, if $k\in (\sigma _Qq)^{-1}(\widehat{Q})$ then $k\in p^{-1}({\widehat{Q}})$ and so (15) holds. Since ${\widehat{Q}}\subset B(Q,l(Q)/8)$, Lemma 4.4 item 4 implies (16).

Now let $R\in {\mathcal {C}}'$ with $l(R)\le l(Q)$ and $k\in p^{-1}({\widehat{Q}} \cap {\widehat{R}})$. Then (18) for *R* implies

$$\begin{aligned} \Vert p_k-q_{\sigma _R(k)}\Vert < \frac{l(R)}{24m} \le \frac{l(Q)}{24m} \end{aligned}$$

and so (12) implies $q_{\sigma _R(k)}\in \widehat{Q}$. The similar argument with *p* and $\sigma _R q$ exchanged gives (17). $\square$

By carefully partitioning *E* using the ${\widehat{Q}}$, we use Lemma 4.9 to construct the desired permutation on $p^{-1}(E)$.

#### Proposition 4.10

There exists a bijection $\tau :p^{-1}(E)\rightarrow q^{-1}(E)$ such that

$$\begin{aligned} \sum _{k\in p^{-1}(E)}\Vert p_k-q_{\tau (k)}\Vert ^2 \le \sum _{Q\in {\mathcal {C}}'}W_2(\phi ^*_Q(p),\phi ^*_Q(q))^2. \end{aligned}$$

#### Proof

Let

$$\begin{aligned} {\mathcal {C}}''=\{Q\in {\mathcal {C}}': p^{-1}({\widehat{Q}}) \ne \emptyset \}. \end{aligned}$$

Note that, by (15), ${\mathcal {C}}''$ can equivalently be defined as the set of $Q\in {\mathcal {C}}$ with $q^{-1}({\widehat{Q}}) \ne \emptyset$. Since ${\mathcal {C}}''$ is finite, we enumerate it as

$$\begin{aligned} {\mathcal {C}}'' = \{Q_1,Q_2,\ldots ,Q_j\} \end{aligned}$$

in such a way that

$$\begin{aligned} l(Q_1) \ge l(Q_2) \ge \cdots \ge l(Q_j). \end{aligned}$$

Then, for $1\le i\le k\le j$, applying Lemma 4.9 with $Q=Q_k$ and $R=Q_i$ gives

19 $$\begin{aligned} p^{-1}\left( {\widehat{Q}}_i \cap {\widehat{Q}}_k\right) = (\sigma _{Q_k} q)^{-1}\left( {\widehat{Q}}_i \cap {\widehat{Q}}_k\right) \quad \forall 1\le i \le k \le j. \end{aligned}$$

Let $B_1 = {\widehat{Q}}_1$ and for each $2\le k\le j$ define

$$\begin{aligned} B_k:= {\widehat{Q}}_k {\setminus } \bigcup _{i=1}^{k-1} \widehat{Q}_i = {\widehat{Q}}_k {\setminus } \bigcup _{i=1}^{k-1} \widehat{Q}_i \cap {\widehat{Q}}_k. \end{aligned}$$

Then (19) implies that $\sigma _{Q_k}$ is a permutation between $p^{-1}(B_k)$ and $\sigma _{Q_k}q^{-1}(B_k)$ for each $1\le k\le j$. Therefore, we define a bijection

$$\begin{aligned} \tau :p^{-1}(E) \rightarrow q^{-1}(E) \end{aligned}$$

by setting $\tau$ to equal $\sigma _{Q_k}$ on $D_k:=p^{-1}(B_k)$ for each $1\le k\le j$. Then

$$\begin{aligned} \sum _{k\in p^{-1}(E)} \Vert p_k-q_{\tau (k)}\Vert ^2&= \sum _{i=1}^j\sum _{k\in D_i} \Vert p_k-q_{\tau (k)}\Vert ^2 \\&= \sum _{i=1}^j\sum _{k\in D_i} \Vert p_k-q_{\sigma _{Q_i}(k)}\Vert ^2 \\&= \sum _{i=1}^j\sum _{k\in D_i} \Vert \phi _{Q_i}(p_k)-\phi _{ Q_i}(q_{\sigma _{Q_i}(k)})\Vert ^2 \\&\le \sum _{Q\in {\mathcal {C}}''}\sum _{k=1}^m \Vert \phi _{Q}(p_k)-\phi _{ Q}(q_{\sigma _{Q}(k)})\Vert ^2, \end{aligned}$$

using (16) for the third equality. Finally (11) completes the proof. $\square$

Next we consider the points outside *E* for which we use the distance to $\partial \Omega$ to estimate $\delta$.

#### Lemma 4.11

For any bijection

$$\begin{aligned} \sigma :p^{-1}(\Omega {\setminus } E) \rightarrow q^{-1}(\Omega {\setminus } E) \end{aligned}$$

we have

$$\begin{aligned} \sum _{k\in p^{-1}(\Omega {\setminus } E)} ({\text {dist}}(p_k,\partial \Omega ) + {\text {dist}}(q_{\sigma (k)},\partial \Omega ))^2 \le m^3 c_2 \sum _{Q\in {\mathcal {C}}{\setminus } {\mathcal {C}}'} W_2(\phi ^*_Q(p),\phi ^*_Q(q))^2, \end{aligned}$$

for $c_2\ge 1$ that depends only upon *n*.

#### Proof

For a moment fix $k\in p^{-1}(\Omega {\setminus } E)$ and let $Q\in {\mathcal {C}}$ contain $p_k$. Then necessarily $Q\not \in {\mathcal {C}}'$. Therefore (13) and (6) imply

$$\begin{aligned} W_2(\phi ^*_Q(p),\phi ^*_Q(q)) \ge \frac{c_1}{m}l(Q) \ge \frac{c_1}{5\sqrt{n}m} {\text {dist}}(p_k,\partial \Omega ). \end{aligned}$$

Since each $Q\in {\mathcal {C}}$ contains at most *m* such points $p_k$,

$$\begin{aligned} \sum _{k\in p^{-1}(\Omega {\setminus } E)} {\text {dist}}(p_k,\partial \Omega )^2&\le \frac{25 m^2 n}{c_1^2} m \sum _{Q\in {\mathcal {C}}{\setminus } {\mathcal {C}}'} W_2(\phi ^*_Q(p),\phi ^*_Q(q))^2. \end{aligned}$$

The same estimate for $\sigma q$ gives the desired inequality for $c_2=4\cdot 25 n/c_1^2$. $\square$

We combine our previous results to show that $\phi ^*_Q$ is a bi-Lipschitz embedding.

#### Theorem 4.12

For any $$p,q\in \mathcal {A}_m(\Omega ^*)$$,

$$\begin{aligned} \frac{W_2(p,q)^2}{c_3 m^3} \le \sum _{Q\in {\mathcal {C}}} W_2(\phi ^*_Q(p),\phi ^*_Q(q))^2 \le c_3W_2(p,q)^2, \end{aligned}$$

where $c_3\ge 1$ depends only upon *n*.

#### Proof

The right hand inequality is given by Lemma 4.7.

For the left hand inequality, let $\tau$ be the bijection obtained from Proposition 4.10 and arbitrarily extend it to a bijection of $\{1,\ldots ,m\}$. Then

$$\begin{aligned} \sum _{Q\in {\mathcal {C}}} W_2(\phi ^*_Q(p),\phi ^*_Q(q))^2&= \sum _{Q\in {\mathcal {C}}'}\sum _{k=1}^m \Vert \phi _{Q}(p_k)-\phi _{ Q}(q_{\sigma _{Q}(k)})\Vert ^2 \\&\quad + \sum _{Q\not \in {\mathcal {C}}'}\sum _{k=1}^m \Vert \phi _{Q}(p_k)-\phi _{ Q}(q_{\sigma _{Q}(k)})\Vert ^2 \\&\ge \sum _{k\in p^{-1}(E)} \Vert p_k-q_{\tau (k)}\Vert ^2\\&\quad + \frac{1}{c_2 m^3}\sum _{k\in p^{-1}(\Omega {\setminus } E)} ({\text {dist}}(p_k,\partial \Omega ) + {\text {dist}}(q_{\tau (k)},\partial \Omega ))^2\\&\ge \frac{1}{c_2 m^3} \sum _{k=1}^{m}\delta (p_k,q_{\tau (k)})^2\\&\ge \frac{1}{c_2 m^3} W_2(p,q)^2, \end{aligned}$$

using Proposition 4.10 and Lemma 4.11 for the first inequality. $\square$

## The embedding into Hilbert space

In this section we conclude the proof of Theorem 1.1. Let $\xi :\mathcal {A}_m({\mathbb {R}}^{n+1}) \rightarrow {\mathbb {R}}^N$ be the embedding given by Theorem 2.3. We write

$$\begin{aligned} \ell _2 = \sum _{Q\in {\mathcal {C}}} {\mathbb {R}}^N \end{aligned}$$

as a direct $l_2$-sum over ${\mathcal {C}}$. Recall the construction of ${\mathcal {T}}$ from Definition 4.1.

### Lemma 5.1

The function $\xi ':{\mathcal {T}}\rightarrow \ell _2$ defined by

$$\begin{aligned} \sum _{Q\in {\mathcal {C}}} \mathcal {A}_m({\mathbb {R}}^{n+1})&\rightarrow \sum _{Q\in {\mathcal {C}}} {\mathbb {R}}^N \\ \xi ' = \sum _{Q\in {\mathcal {C}}} \xi \end{aligned}$$

is well defined. Moreover, for any $a,b\in {\mathcal {T}}$,

$$\begin{aligned} \frac{1}{c m^{2n+2}} \sum _{Q\in {\mathcal {C}}} W_2(a_Q,b_Q)^2 \le \Vert \xi '(a)-\xi '(b)\Vert ^2 \le \sum _{Q\in {\mathcal {C}}} W_2(a_Q,b_Q)^2, \end{aligned}$$

for $c\ge 1$ depending only upon *n*.

### Proof

Let $a\in {\mathcal {T}}$, so that

$$\begin{aligned} \sum _{Q\in {\mathcal {C}}} W_2(p_Q,0)^2 <\infty . \end{aligned}$$

Since $\xi$ is 1-Lipschitz this implies that

$$\begin{aligned} \sum _{Q\in {\mathcal {C}}} \Vert \xi (p_Q)\Vert ^2 = \sum _{Q\in {\mathcal {C}}} \Vert \xi (p_Q)-\xi (0)\Vert ^2 \le \sum _{Q\in {\mathcal {C}}} W_2(p_Q,0)^2 <\infty . \end{aligned}$$

Hence, $\xi '$ is well defined. Moreover, using that $\xi$ is 1-Lipschitz again, we have, for any $b\in {\mathcal {T}}$,

$$\begin{aligned} \sum _{Q\in {\mathcal {C}}} \Vert \xi (a_Q)-\xi (b_Q)\Vert ^2 \le \sum _{Q\in {\mathcal {C}}} W_2(a_Q,b_Q)^2, \end{aligned}$$

so that $\xi '$ is also 1-Lipschitz. Finally, Theorem 2.3 gives

$$\begin{aligned} \sum _{Q\in {\mathcal {C}}} \Vert \xi (a_Q)-\xi (b_Q)\Vert ^2 \ge \frac{1}{cm^{2n+2}}\sum _{Q\in {\mathcal {C}}} W_2(a_Q,b_Q)^2. \end{aligned}$$

$$\square $$

### Theorem 5.2

There exists a bi-Lipschitz embedding $\zeta :\mathcal {B}_m(\Omega )\rightarrow \ell _2$ with distortion at most $cm^{n+5/2}$, for $c\ge 1$ depending only upon *n*. That is, for any $p,q\in \mathcal {B}_m(\Omega )$,

$$\begin{aligned} \frac{W_2(p,q)}{c m^{n+5/2}} \le \Vert \zeta (p)-\zeta (q)\Vert \le c W_2(p,q). \end{aligned}$$

### Proof

First isometrically embed $\mathcal {B}_m(\Omega )$ into $\mathcal {A}_m(\Omega ^*)$ via Corollary 3.12. One then applies Theorem 4.12 to bi-Lipschitz embed $\mathcal {A}_m(\Omega ^*)$ into ${\mathcal {T}}$. Finally, Lemma 5.1 bi-Lipschitz embeds ${\mathcal {T}}$ into $\ell _2$, as required. $\square$

### Remark 5.3

For $n\ge 3$, the distortion of any embedding of $\mathcal {A}_m(\Omega ^*)$ into $\ell _2$ converges to $\infty$ as $m$ increases. In particular, $Wb_2(\Omega )$ does not bi-Lipschitz embed into $\ell _2$.

Indeed, by Eq. (7) we see that $\mathcal {A}_m(\Omega ^*)$ contains an isometric copy of $A_m(Q)$ for some cube *Q*. Thus, the distortion of any embedding into $\ell _2$ is at least that of $\mathcal {A}_m(Q)$. For $n\ge 3$, Andoni, Naor and Nieman [4, Theorem 7] prove that $W_2({\mathbb {R}}^n)$ does not coarsely, in particular bi-Lipschitz, embed into any Banach space of non-trivial type, namely Hilbert space. Since the set of discrete measures is dense in $W_2({\mathbb {R}}^n)$, a scaling argument shows that the distortion of any bi-Lipschitz embedding of $\mathcal {A}_m(Q)$ must converge to $\infty$ as $m$ does.

The same conclusion can be made for $n=2$ using an unpublished result of Austin and Naor announced in [4, Remark 8], which states that $W_2({\mathbb {R}}^2)$ does not bi-Lipschitz embed into $L_1$ and, hence, does not bi-Lipschitz embed into $\ell _2$.

## Data Availability

There is no data.

## Footnotes

1 We adopt the standard convention that Hilbert space is the unique complete and separable infinite dimensional inner product space, up to isometric isomorphism.
